# A Least Angle Regression Model for the Prediction of Canonical and Non-Canonical miRNA-mRNA Interactions

**DOI:** 10.1371/journal.pone.0040634

**Published:** 2012-07-17

**Authors:** Julia C. Engelmann, Rainer Spang

**Affiliations:** Department of Statistical Bioinformatics, Institute for Functional Genomics, University of Regensburg, Regensburg, Germany; Queen’s University Belfast, United Kingdom

## Abstract

microRNAs (miRNAs) are short non-coding RNAs with regulatory functions in various biological processes including cell differentiation, development and oncogenic transformation. They can bind to mRNA transcripts of protein-coding genes and repress their translation or lead to mRNA degradation. Conversely, the transcription of miRNAs is regulated by proteins including transcription factors, co-factors, and messenger molecules in signaling pathways, yielding a bidirectional regulatory network of gene and miRNA expression. We describe here a least angle regression approach for uncovering the functional interplay of gene and miRNA regulation based on paired gene and miRNA expression profiles. First, we show that gene expression profiles can indeed be reconstructed from the expression profiles of miRNAs predicted to be regulating the specific gene. Second, we propose a two-step model where in the first step, sequence information is used to constrain the possible set of regulating miRNAs and in the second step, this constraint is relaxed to find regulating miRNAs that do not rely on perfect seed binding. Finally, a bidirectional network comprised of miRNAs regulating genes and genes regulating miRNAs is built from our previous regulatory predictions. After applying the method to a human cancer cell line data set, an analysis of the underlying network reveals miRNAs known to be associated with cancer when dysregulated are predictors of genes with functions in apoptosis. Among the predicted and newly identified targets that lack a classical miRNA seed binding site of a specific oncomir, miR-19b-1, we found an over-representation of genes with functions in apoptosis, which is in accordance with the previous finding that this miRNA is the key oncogenic factor in the mir-17-92 cluster. In addition, we found genes involved in DNA recombination and repair that underline its importance in maintaining the integrity of the cell.

## Introduction

miRNAs are small endogenous RNAs with a length of about 22 nt with gene regulatory functions and are found in plants and animals [1]. Unlike other classes of small RNAs, miRNAs undergo a characteristic biogenesis which consists of a transcript folding back on itself to form a distinctive hairpin structure [2]. After processing, miRNAs form a complex with an Argonaute protein, pair with the target mRNA and induce post-transcriptional repression of the gene product [1]. Since more than half of the human protein-coding genes seem to have conserved miRNA pairing sites in their 3′-UTR [3], it is difficult to find a biological process or pathway which is not at all influenced by regulation from miRNAs [1]. It is most likely that the interactions of miRNAs and mRNAs are context-specific as miRNAs are known to play important roles in differentiation, development, cancer and more [4]–[8]. Knowing which miRNA regulates which gene at a certain time and location is crucial not only to understand gene regulation but also for a systems biology account of the cell. Two mechanisms of post-translational repression of mRNAs by miRNAs are well-described for metazoans: 1) at sites with high complementarity between mRNA and miRNA, a miRNA can bind to the mRNA and induce mRNA cleavage with the help of an Argonaute protein [1], [9]; 2) the miRNA induces translational repression or mRNA destabilization, e.g., by inhibition of translation initiation and poly(A) shortening, or both [1], [10]. In animals, the second mechanism, which requires less sequence complementarity between mRNA and miRNA, is used more often [1].

Approaches to elucidate miRNA-mRNA associations can be classified into two principal classes: 1) solely sequence-based approaches and 2) expression data-based approaches which often include sequence features. While the sequence-based approaches focus on one-to-one relationships, the data-based methods are more flexible to also search for many-to-one or one-to-many relationships. The sequence-based miRNA target prediction algorithms focus on predicting direct targets of miRNAs based on sequence similarities, especially the seed sequence, and evolutionary conservation. The target prediction problem is hard and the prediction accuracy is currently still low. The shortness of seed sequences leads to high numbers of false positive predictions [1] and low sensitivity. State-of-the-art target predictors include features additional to the seed region in the 3′-UTR of the mRNA, e.g., conservation of the site across related species [3], [11], [12]. However, this seems to be insufficient in reducing the number of false-positives, plus, in the case of sequence conservation, misses species-specific poorly conserved candidate sites.

Beyond sequence complementarity, Grimson and co-authors [13] report five features of site context that improve binding site efficacy and others have also reported on the impact of structural factors on target recognition [14], [15]. Very recently, additional mechanisms of miRNA-mRNA interactions have been shown to affect mRNA expression levels [16]. While Elefant et al. showed that seed pairing is the dominant mechanism in down-regulating miRNA target genes (81% of binding sites), they also found predicted functional 3′-compensatory binding sites in a considerable fraction of target genes (18.6%). 3′-compensatory binding sites are characterized by insufficient 5′ seed pairing which is compensated by extensive 3′ end pairing. An additional 0.5% of down-regulated targets were predicted to host centered sites. These sites lack both perfect seed pairing and 3′-compensatory binding, but allow for extensive pairing (11–12 contiguous pairs) at the center of the miRNA. Non-canonical binding mechanisms are not yet considered by current miRNA target prediction algorithms and thus raise hope that prediction accuracies will improve in the near future.

Expression data-based approaches usually start from high dimensional miRNA and mRNA expression data. While mere negative correlations do recover some miRNA-mRNA regulatory relationships, more powerful approaches have been developed to make use of paired mRNA-miRNA expression profiles [17], [18]. Huang et al. proposed a Bayesian model based on predicted miRNA targets from TargetScan and miRNA and mRNA expression data, which tries to account for mRNA expression given the miRNA expression. This comes down to a feature selection problem in determining the miRNAs which best predict the observed mRNA expression profile. A similar approach is taken by the LASSO, and indeed it has also been shown to be a valuable approach for deriving functional miRNA-mRNA interactions from expression data, outperforming plain correlations [19].

Another elegant approach has been proposed by Betel et al. [20]. Therein, support vector regression is used to score the local and global context of a miRNA binding site after having been trained on miRNA transfection experiments. The algorithm can predict non-canonical (lacking perfect seed binding), non-evolutionary conserved sites and allows for multiple regulators of the same gene. However, it requires a negative association between miRNA and mRNA and needs experimental data for training.

Even in the absence of miRNA expression data, Radfar et al. [21] showed that intronic miRNA expression can be inferred from host gene expression. They classify miRNAs into the ones tightly co-regulated with their host gene, those transcribed from the same promotor but the mRNA itself is targeted by one or several miRNAs and those which have independent promotors.

Two advantages of the expression data-based, and expression data- and sequence integrating approaches are a) they allow to study regulatory feedback, and b) they can account for multiple regulators for the same gene. With respect to a), miRNAs regulate the expression of genes via mediating the degradation or translational repression of mRNAs. Vice versa the expression of the miRNAs themselves is under the control of genes like transcription factors and their mediators. Together this yields a bidirectional regulatory interplay of gene expression and miRNA expression. Complementary target binding based on sequence features can at most explain one direction. With respect to b), regulators often work in teams. Early computational approaches to uncover regulation of gene expression by miRNAs on a large scale have focused on one-to-one relationships between a gene (mRNA) and a miRNA [22]–[24]. This simplification does not reflect the true state of nature. It is known that one miRNA can regulate the expression of several genes that have the same miRNA binding site in their 3′-UTR, and that some genes carry predicted binding sites for more than one miRNA [25]. Therefore it is likely that these genes are also regulated by more than one miRNA. Whether several miRNAs regulate the expression of a gene in concert or whether individual miRNAs are used context- and/or time- specific has not yet been studied extensively. More recent approaches consider multiple miRNAs regulating the same gene [18], [19].

Two further points remain to be addressed: a) miRNAs can also increase gene expression. While most research focused on regulation where the miRNA decreases the amount of mRNA or protein, there is evidence that a miRNA can also increase the amount of mRNA or protein, e.g in quiescent cells, while in proliferating cells they are more likely to repress translation [26], [27]. Current approaches neglect this possibility and restrict themselves to negative associations of miRNAs and mRNAs. b) Indirect regulation. Besides the direct interaction of a miRNA and a gene, a miRNA can also act on an intermediate regulatory molecule which then affects a functional target mRNA. Thus, increased miRNA levels may lead to repression or elevation of gene expression levels when intermediate players are involved. This type of regulation is also neglected when focusing on negative interactions. Therefore, data-driven methods to identify functional miRNA-mRNA interactions should not only focus on perfect seed pairing and negative associations but allow for the full spectrum of regulatory mechanisms.

In this manuscript, we first show that entire mRNA expression profiles or large parts of them can be reconstructed only from the expression of miRNAs and vice versa, a precondition that has not been shown in previous regression based approaches. Next, we propose a two-step computational model that in its first step uses binding site information from miRNA target prediction algorithms (TargetScan [3], microCosm [12], PicTar [28], and DIANA/microT [29]) and in the second step, we release this constraint and allow all remaining miRNAs as predictors of gene expression. In this procedure, we give preference to the miRNAs having a matching seed sequence, but also allow for mechanisms that do not rely on perfect seed binding. Thus we are able to discover new interactions which would be overlooked by data-driven computational models described so far. In addition, we also compute a reverse model where genes predict the expression of miRNAs, which allows to build bi-directional networks of miRNA-mRNA regulation.

## Results

### Predicting Gene from miRNA Expression

We exemplify our method using the NCI-60 panel of human cancer cell lines. It holds 59 samples for which both gene and miRNA expression profiles are available. After a filtering step to remove genes and miRNAs that vary little across samples, we used 311 miRNA expression levels and 4,878 gene expression levels to study the functional interactions between both types of molecules.

First, we identified miRNA predictors for the expression of all 4,878 genes in the analysis. We applied least angle regression (LARS) [30], a computationally efficient method that combines predictive linear regression in high dimensions with the selection of small sets of predictors. Independently for all 4,878 genes, one regression model was learned using all 311 miRNAs as possible predictors. We generated predictions of gene expression in 10-fold cross-validation, and recorded mean squared errors (cv-MSE) along with all miRNAs identified as predictors.

The procedure produces regression models and lists of candidate predictors for all mRNAs. However, some of the regression models have a poor predictive performance in cross-validation. In order to identify genes whose expression can be predicted only from miRNA expression we contrasted observed prediction errors (cv-MSEs) with simulated distributions of cv-MSEs that arise, if the expression profiles are not properly paired. We did this by running the regression analysis on data where the samples are randomly permuted in one data set such that "paire" samples no longer stem from the same cell line. In other words, we predicted the expression profile of a cell line from the miRNA profile of another cell line, which can only result in random predictions. For every predicted gene, we learned 100 models with permuted pairings yielding 487,800 randomized models. The cv-MSE was recorded across the 100 permutations and an empirical p-value was calculated by counting the relative frequency of permutation based cv-MSEs that were smaller than the cv-MSE of the correctly paired data.

False Discovery Rates (FDR) were estimated from the distributions of p-values [31] and a list of top ranking models with FDR <0.01 was considered for further analysis. This resulted in a total of 928 genes whose expression could be predicted from miRNA expression alone (File S1). More statistical details of the regression modeling and permutation testing are given in the Methods section below.

**Figure 1:**
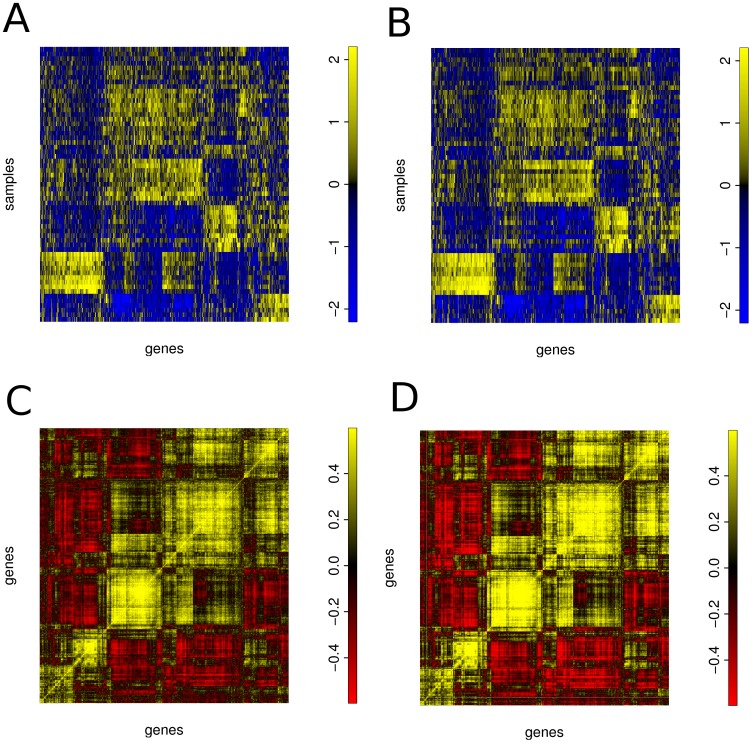
Gene expression can be predicted from miRNA expression with high accuracy. A) Gene expression values. B) Gene expression values predicted from miRNA expression. C) Correlation of gene expression values. D) Correlation of predicted gene expression values. Besides the gene expression structure, the correlation structure is well-preserved. Predictions are from unconstrained gene models. In subfigures A and B, samples are in rows and genes are in columns. Expression values were centered and scaled and color-coded with blue representing low and yellow high expression values. The subfigures C and D show the gene-by-gene correlation structure of the genes displayed in subfigures A and B. Here, yellow indicates high correlation and red indicates anti-correlation of genes. The order of genes and samples is the same for all subfigures.

**Figure 2:**
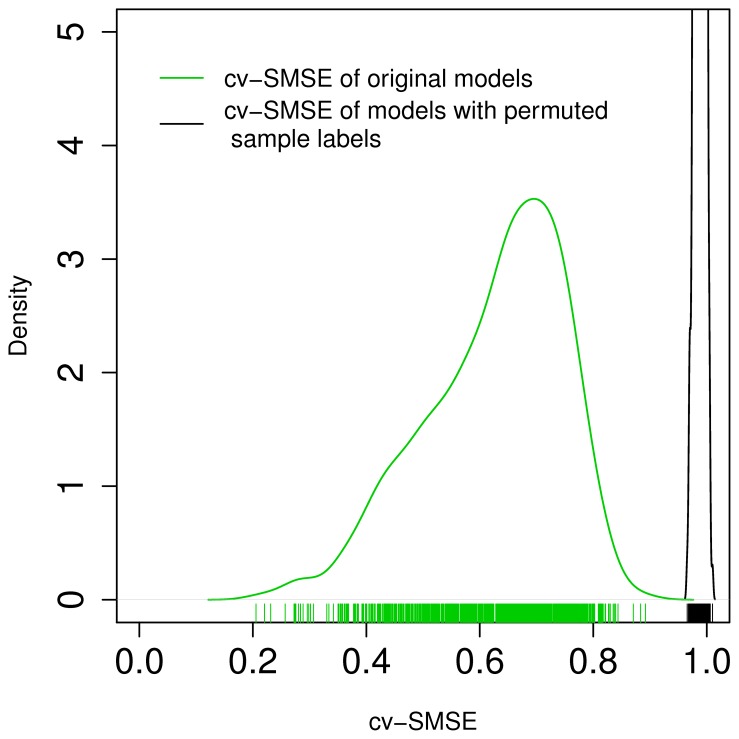
Scaled mean squared cross-validated prediction error (cv-SMSE) of unconstrained models with correct sample lables (green) and of models with permuted sample labels (black). The range of the cv-SMSE is shown on the horizontal axis, while the density is shown on the vertical axis. The lines indicate the density of the data distributions, and small vertical stripes display individual data points.

Since regression is a prediction algorithm, and least angle regression tackles the variable selection problem, we first check its prediction and later the variable selection capabilities. Figures 1 (A) and 1 (B) contrast the observed expression values of the 928 predictable genes with their cross-validation predictions from the miRNA data. Not only do we see a good agreement of predictions and observations for each gene individually but also a good agreement of the clustering structure between measured and predicted gene expression values. This is further supported by Figures 1 (C) and 1 (D) that contrast all pairwise correlation coefficients across measured expression values (left) and predicted expression values (right). This is remarkable, because the regression models have been learned for all predicted genes independently from each other. A more quantitative assessment of prediction accuracies is given in Figure 2. It shows the distribution of mean squared cross validation prediction errors across genes. To make the cv-MSE comparable across genes, it was divided by the variance of the respective gene, yielding a scaled mean squared prediction error (cv-SMSE). The figure shows distributions of the cv-SMSE of the original models (green line) and that of the corresponding permuted models (black line), while the individual cv-SMSE values are shown as green respectively black vertical stripes below the density curves. For the prediction accuracy see also Table 1 where we refer to the models as unconstrained models.

**Table 1:** Performance of the different types of gene and miRNA models.

Gene models	number of sign.models	cv-SMSE (0.05, 0.5,0.95 quantile)	difference in r (0.05and 0.95 quantile)	number of predictors, median
Unconstrained	928	0.3998 0.6488 0.7871	−0.1719 0.1533	2–55; 31
Direct target (DTM)	480	0.5739 0.7637 0.8743	−0.3541 0.3552	1–45; 6
Residual	581	0.3564 0.6305 0.7903	−0.1641 0.1687	1–57; 29
Negative regulation and direct target	716	0.6777 0.8936 0.9717	−0.4552 0.4073	1–26; 3
Negative regulation, residual	689	0.2052 0.7039 3.9824	−0.6058 0.5700	1–54; 24
miRNA model, unconstrained	93	0.3493 0.5844 0.7872	−0.1099 0.1139	8–54; 37

Number of significant models, scaled mean squared prediction error from cross-validation (cv-SMSE), 0.05 and 0.95 quantile of the differences in gene-wise correlation between original and predicted gene expression, and range and median of number of predictors for each model type.

Interestingly, the expression of none of the genes could be optimally predicted by a single miRNA. For all 928 predictable genes, combinations of several miRNAs (at least 2) improved prediction emphasizing the extent and importance of functional miRNA-mRNA interactions in gene regulation.

### Integration of Target Sequence Information

Using predictions from four different algorithms (microCosm [12], TargetScan [3], DIANA/microT, [29], and doRiNA (formerly PicTar) [28]), we searched for miRNA-gene interactions predicted by at least two of the four algorithms in our models. Only 4.9% of predictors included a target sequence. The observation suggests that functional gene regulation through miRNAs can not be reduced to direct miRNA-mRNA interactions. However, this observation may also result from modeling limitations. The expression of modules of miRNAs strongly correlate suggesting that the expression of these miRNAs themselves are jointly regulated. In the context of LARS regression this can lead to the replacement of a functionally regulating miRNA by a co-expressed but functionally uninvolved miRNA as predictor [32]. To follow up on this problem, we integrated sequence based target information in our analysis by allowing only miRNAs with an annotated target sequence for the predicted genes as possible predictors. For 4,820 of the 4,878 selected genes, we found at least one miRNA with a complementary target sequence. The dimensionality of the regression problems now strongly varies across predicted genes ranging from only one miRNA with a matching target sequence to 261 possible predictors.

The prediction accuracy of the constrained models was lower than for the unconstrained models, see Table 1, where we refer to the models as direct target models (DTM). However, we still found 480 genes for which the constrained models yielded significant predictions (File S2). Interestingly, only 4.8% of genes were optimally predicted with a single miRNA, for all others complementing expression information from several miRNAs increased cross-validated prediction accuracy. The significant DTMs suggest that in many cases the correlation between gene and miRNA expression can be attributed to direct interactions. However, the data also suggests that many miRNAs operate in concert.

### Identification of New Regulators

The reduced predictive performance also indicates that important predictive information is missed. This is further supported by a third set of models that is trained on all miRNAs except those used in the DTMs. These residual models are not used to predict the measured gene expression values but to predict the residuals of the DTM. Our intention is to find complementary predictors to the miRNAs that contain a matching target sequence. Table 1 indicates that again we found 581 models with significant predictions of residuals, or gene expression values if no targeting miRNAs were reported for the gene in the database. This second step allows for the identification of new regulating miRNAs, which have not yet been reported to regulate the response mRNA, and thus creates hypotheses on so far unknown regulators. Note that the combination of DTMs and residual models does not reflect the same information as the unconstrained models. While the predictors of the unconstrained models replace direct target miRNAs, the predictors of the residual models complement the predictive information held in the predictors of the direct target models. In the supporting information section we provide a list of *de novo* identified candidate regulator miRNAs for all 581 genes with significant residual models (File S3).

### Focusing on Negative Regulatory Interactions

In the prevalent conception of miRNA-mediated gene regulation, the miRNA is negatively regulating the expression of the gene. This should be reflected in regression models by negative regression coefficients. However, in all models discussed so far we observed both positive and negative coefficients. This might in part be explained by the observation of Vasudevan and coworkers [26], [27], who report that a miRNA can also increase the amount of mRNA or protein, and by indirect regulation where an intermediate regulator is negatively affected by the miRNA which then leads to an increase of mRNA levels.

However, predictive information does not always reflect that a miRNA regulates a gene. It can also arise when a gene is co-expressed with a miRNA. While the second mechanism is functionally as important as the first, one might still want to focus on miRNA mediated regulation. This can be done by only allowing negative regression coefficients in the direct target models, yielding a fourth set of models that we call negative regulation models (NRM). We found 716 significant NRM with a predictive accuracy that is comparable to that of the original DTM (Table 1, File S4). Also for the NRM we learned residual models and provide candidate lists of newly identified functional regulator-target interactions in the supporting information section (File S5).

### Functional Targets of Oncogenic miRNAs

The NCI-60 data set consists of human cell lines originating from different cancers. Therefore, we expected to find functional interactions between oncogenic miRNAs and genes involved in the genesis and progression of cancer. For a selection of 13 miRNAs with known functions in cancer development described by Esquela-Kerscher [33], we selected significant models from the negative regulation and residual models described above where at least one of these onco-miRNAs served as a predictor. Interestingly, on their own these miRNAs did not predict the expression of their potential targets. This was only accomplished in concert with other miRNAs. These complementing predictors are newly identified candidates of oncogenic miRNAs. They are listed in the supporting information section (File S5).

We tested the list of genes that were predicted to be regulated by individual onco-miRNAs from the negative regulation and residual models for enrichment of Gene Ontology terms [34]. The results are summarized in Table 2. Most interestingly, genes that are involved in programmed cell death, regulation of the apoptotic process and other processes that play a role in tumor development are significantly over-represented among the predicted functional targets of many onco-miRNAs, reinforcing their association with tumorigenesis. Table 2 summarizes enrichment of GO terms for the predicted targets of all onco-miRNAs.

**Table 2:** Most frequent GO terms over-represented in oncomir target models.

GOBPID	median p-value	median odds-ratio	median expected count	median count	size	term	oncomirs
GO:0012501	0.0045	2.91	6.37	13.5	164	**programmed cell death**	mir-15a-2, mir-19b-1
GO:0016265	0.0084	2.84	6.91	14	178	**death**	mir-15a-2, mir-19b-1
GO:0042981	0.0113	2.7	4.93	10.5	127	**regulation of apoptotic process**	mir-15a-2, mir-19b-1
GO:0045333	0.0191	6.32	0.59	3	17	cellular respiration	let-7a-1, let-7e, let-7i
GO:0006310	0.0202	8.28	0.49	3	14	DNA recombination	mir-19b-1, mir-21
GO:0006163	0.0224	2.97	2.86	7	81	purine nucleotide metabolic process	mir-20, mir-92-1
GO:0070727	0.0234	4.33	2.12	6.5	97	cellular macromolecule localization	let-7d-v1, mir-145
GO:0008104	0.0241	3.06	4.03	9	171	protein localization	let-7d, mir-145
GO:0048562	0.0265	5.53	0.67	3	18	embryonic organ morphogenesis	mir-92-1, mir-145
GO:0022613	0.0275	8.45	0.37	2.5	19	ribonucleoprotein complex biogenesis	let-7f-1, mir-16-1
GO:0000209	0.0292	6.06	0.65	3	19	protein polyubiquitination	let-7f-1, mir-19b-1
GO:0048592	0.0295	6.98	0.46	2.5	14	eye morphogenesis	let-7d-v2, mir-19b-2
GO:0010863	0.0297	10.9	0.27	2	12	positive regulation of phospholipase C activity	let-7a-2, let-7e
GO:0060193	0.0297	10.9	0.27	2	12	positive regulation of lipase activity	let-7a-2, let-7e
GO:0007276	0.0307	4.13	1.19	4	42	gamete generation	let-7d-v2, mir-21-17
GO:0015031	0.0313	2.34	4.57	9	131	protein transport	let-7d-v2, let-7i, mir-145
GO:0071705	0.0317	4.48	1.26	4	12	nitrogen compound transport	mir-142, mir-143
GO:0032870	0.0318	3.58	1.48	4.5	47	cellular response to hormone stimulus	let-7e, mir-21
GO:0006915	0.0326	3.55	2.25	5.5	161	**apoptotic process**	let-7d-v1, mir-15a
GO:0010942	0.0378	2.26	5.54	10	65	**positive regulation of cell death**	mir-19b-1, mir-155
GO:0007093	0.0383	4.76	0.77	3	16	mitotic cell cycle checkpoint	let-7a-1, mir-19b-1
GO:0010557	0.0386	2.31	4.33	8.5	109	positive regulation of macromolecule biosynthetic process	mir-19b-1, mir-20
GO:0050708	0.0387	7.88	0.32	2	12	regulation of protein secretion	let-7d-v2, mir-15a-2
GO:0055114	0.0421	3.23	1.67	4.5	67	oxidation-reduction process	let-7e, mir-16-1
GO:0009749	0.0431	6.88	0.36	2	11	response to glucose stimulus	let-7e, let-7f, mir-21, mir-155
GO:0034284	0.0431	6.88	0.36	2	11	response to monosaccharide stimulus	let-7e, let-7f, mir-21, mir-155
GO:0034728	0.0465	6.99	0.36	2	11	nucleosome organization	mir-20, mir-21
GO:0006469	0.0474	6.69	0.36	2	19	negative regulation of protein kinase activity	let-7f-1, mir-18

GOBPID stands for the Gene Ontology Biological Process identifier. The median p-value, median odds-ratio, median expected count and median count indicate the median of the respective statistic for this GO term over all significant enrichments of this term in oncomir models. The column count indicates the number of genes of this term observed among oncomir target models. Size is the total number of genes classified with this GO term. Apoptosis-related GO terms are highlighted in bold font.

Among the five most significant Gene Ontology terms which are over-represented in at least two miRNA target gene sets are ‘programmed cell death’, ‘death’, ‘regulation of apoptotic process’ and ‘DNA recombination’. The target gene sets of miR-19b-1 are enriched for four of these terms. This miRNA and its targets are examined in more detail below.

### The Onco-miRNA miR-19b-1

miR-19b-1 belongs to the mir-17-92 cluster which is frequently amplified and over-expressed in lymphomas [33]. miR-19b has been shown to be the key oncogenic miRNA within the mir-17-92 cluster. It is both necessary and sufficient to promote c-myc induced B-cell lymphomagenesis through the repression of apoptosis [35].

We identified 9 genes where miR-19b-1 is one of possibly many predictors in models that have either positive or negative coefficients and predictors with complementary seed sequences (direct target models). Interestingly, in most cases, miR-19b-1 was not chosen as a single predictor but as a co-predictor that needs to be complemented with further miRNAs in order to predict gene expression.

**Figure 3:**
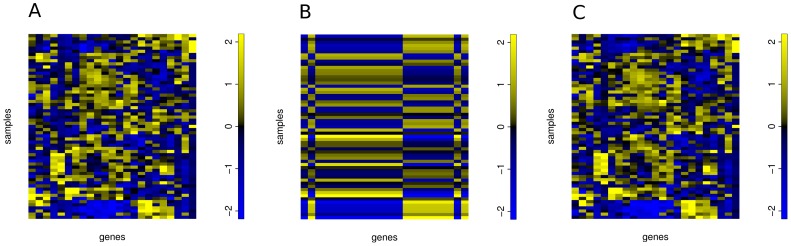
Gene expression of genes for which miR-19b-1 serves as one of the predictors. A) Gene expression values. B) Gene expression predicted from miR-19b-1 only. C) Gene expression predicted from all predictors of the gene models. Gene expression can only be predicted if all predictors of the direct target and the residual model are used. If only miR-19b-1 is allowed as a predictor in the LARS model, the prediction is poor. In all subfigures, samples are in rows and genes in columns. Expression and predicted expression values were centered and scaled and color-coded with blue representing low and yellow high expression values. The order of genes and samples is the same for all sub-figures.

In models that were learned on the residuals of the direct target models, we found 14 genes with miR-19b-1 in their list of predictors, suggesting that the prominent role of miR-19b-1 in oncogenesis might not be restricted to its direct action as a silencing miRNA. This also becomes apparent in Figure 3 that compares the observed expression of functional miR-19b-1 targets (left) to their predicted expression using only miR-19b-1 as predictor (middle) and predictions using miR-19b-1 together with the identified complementary miRNA predictors (right). Whereas the prediction of gene expression values with miR-19b-1 alone is very poor, the prediction using all predictors from the regression models is good.

### Bidirectional Regulatory Networks

Until now, we focused on the regulation of genes via the expression of miRNAs. The expression of the miRNAs themselves is under the control of genes, such as transcription factors and their mediators. Together this yields a bidirectional regulatory interplay of gene expression and miRNA expression.

We complemented our analysis by running the same analysis to predict miRNA expression from mRNA expression, yielding modules of mRNAs as predictors of miRNA expression. We identified 93 miRNAs whose expression could be significantly predicted from gene expression (File S6). The reverse models also exploit the joint information of multiple predictors. A summary of the prediction performance of the reverse models is given in Table 1.

Restricting gene models to the direct target and residual models where only negative coefficients are permitted and leaving the reverse models (genes predicting miRNA expression) unconstrained, yields a bidirectional regulatory network comprised of 2,985 nodes and 23,513 edges in total. This large network can be best browsed by focusing on subnetworks centered around specific genes or miRNAs.

Focusing on the subnetwork around miR-19b-1, some of the genes for which miR-19b-1 serves as a predictor are known to have binding sites (11 genes), but the majority of the genes were *de novo* identified (50 genes).

**Figure 4:**
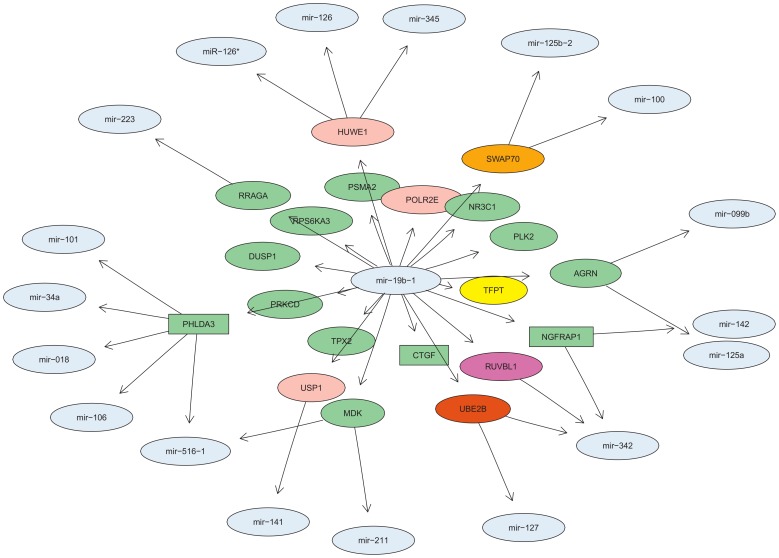
Interaction network of miR-19b-1. Interactions of miR-19b-1 with genes with functions in ‘DNA recombination’ (orange), ‘DNA repair’ (pink), ‘programmed cell death’ (green), all three categories (yellow), in ‘DNA recombination’ and ‘DNA repair’ (purple) and in ‘DNA recombination’ and ‘programmed cell death’ (red). miR-19b-1 is located in the center of the network, around it are genes for which miR-19b-1 is one of the predictors from the negative restricted models (NRM) and the third layer consists of miRNAs for which the genes from the second layer are predictors (unconstrained models). Genes from direct target NRM models (miR-19b-1 is predicted to target the gene by at least two miRNA target prediction algorithms) are represented by rectangular nodes. Genes from residual models have ellipsoid nodes. TFPT: TCF3 (E2A) fusion partner (in childhood Leukemia) [HGNC:13630]; POLR2E: polymerase (RNA) II (DNA directed) polypeptide E, 25 kDa [HGNC:9192]; MDK: midkine (neurite growth-promoting factor 2) [HGNC:6972]; PSMA2: proteasome (prosome, macropain) subunit, alpha type, 2 [HGNC:9531]; SWAP70: SWAP switching B-cell complex 70 kDa subunit [HGNC:17070]; PLK2: polo-like kinase 2 [HGNC:19699]; CTGF: connective tissue growth factor [HGNC:2500]; UBE2B: ubiquitin-conjugating enzyme E2B [HGNC:12473]; NGFRAP1:nerve growth factor receptor (TNFRSF16) associated protein 1 [HGNC:13388]; TPX2: TPX2, microtubule-associated, homolog (Xenopus laevis) [HGNC:1249]; PRKCD: protein kinase C, delta [HGNC:9399]; RUVBL1: RuvB-like 1 (E. coli) [HGNC:10474]; AGRN: agrin [HGNC:329]; NR3C1: nuclear receptor subfamily 3, group C, member 1 (glucocorticoid receptor) [HGNC:7978]; HUWE1: HECT, UBA and WWE domain containing 1, E3 ubiquitin protein ligase [HGNC:30892]; RRAGA: Ras-related GTP binding A [HGNC:16963]; USP1: ubiquitin specific peptidase 1 [HGNC:12607]; RPS6KA3: ribosomal protein S6 kinase, 90 kDa, polypeptide 3 [HGNC:10432]; DUSP1: dual specificity phosphatase 1 [HGNC:3064]; PHLDA3: pleckstrin homology-like domain, family A, member 3 [HGNC:8934].

The three most significant GO terms from the target gene over-representation analysis of miR-19b-1 are ‘DNA recombination’, ‘DNA repair’ and ‘programmed cell death’ (Table 3). This confirms the known role of miR-19b-1 in apoptosis and points to further functions of miR-19b-1 targets in DNA recombination and repair which are also often dysregulated in cancer. Figure 4 shows the subnetwork of genes involved in the three GO categories mentioned above. Interestingly, only three of the genes are predicted to be targets of miR-19b-1 based on sequence information (rectangular nodes), the others are not in the databases (ellipsoid gene nodes) and stem from the residual models. Therefore, the GO term enrichment analysis was driven by the newly identified genes. Among these genes, TFPT (alias FB1, ‘TCF3 (E2A) fusion partner (in childhood Leukemia)’) is annotated with all three of the GO terms selected and a known proto-oncogene in childhood pre-B acute lymphoblastic leukemia [36]. RUVBL1 (alias TIP49) has been shown to be a co-factor of Myc and as such modulates apoptosis in c-Myc-mediated oncogenesis, e.g., in lymphomas [37].

**Table 3:** Most frequent GO terms over-represented in targets of miR-19b-1.

GOBPID	p-value	odds ratio	expected count	count	size	term
GO:0006310	0.0033	8.48	0.67	4	14	DNA recombination
GO:0006281	0.0071	4.96	1.27	5	27	DNA repair
GO:0012501	0.0074	2.37	7.87	15	164	programmed cell death
GO:0006513	0.0128	20.55	0.19	2	4	protein monoubiquitination
GO:0016064	0.0128	20.55	0.19	2	4	immunoglobulin mediated immune response
GO:0016574	0.0128	20.55	0.19	2	4	histone ubiquitination
GO:0022408	0.0128	20.55	0.19	2	4	negative regulation of cell-cell adhesion
GO:0030325	0.0128	20.55	0.19	2	4	adrenal gland development
GO:0051385	0.0128	20.55	0.19	2	4	response to mineralocorticoid stimulus
GO:0000209	0.0130	5.27	0.96	4	20	protein polyubiquitination
GO:0042981	0.0136	2.37	6.10	12	127	regulation of apoptotic process
GO:0016265	0.0158	2.13	8.54	15	178	death
GO:0070507	0.0169	6.94	0.58	3	12	regulation of microtubule cytoskeleton organization
GO:0043525	0.0206	13.69	0.24	2	5	positive regulation of neuron apoptosis
GO:0033554	0.0212	2.28	5.71	11	121	cellular response to stress
GO:0010564	0.0216	3.14	2.26	6	47	regulation of cell cycle process
GO:0010942	0.0289	2.65	3.07	7	65	positive regulation of cell death
GO:0010557	0.0296	2.23	5.23	10	109	positive regulation of macromolecule biosynthetic process
GO:0007052	0.0300	10.25	0.29	2	6	mitotic spindle organization
GO:0031328	0.0315	2.12	6.05	11	126	positive regulation of cellular biosynthetic process
GO:0009893	0.0319	1.96	8.45	14	176	positive regulation of metabolic process
GO:0045935	0.0351	2.15	5.38	10	112	positive regulation of nucleobase-containing compound metabolic process
GO:0052548	0.0366	3.64	1.30	4	27	regulation of endopeptidase activity
GO:0009416	0.0384	8.48	0.33	2	7	response to light stimulus
GO:0002449	0.0407	8.20	0.34	2	7	lymphocyte mediated immunity
GO:0007613	0.0407	8.20	0.34	2	7	memory
GO:0009411	0.0407	8.20	0.34	2	7	response to UV
GO:0007093	0.0441	4.44	0.82	3	17	mitotic cell cycle checkpoint
GO:0033043	0.0468	2.55	2.69	6	56	regulation of organelle organization
GO:0065009	0.0469	1.84	8.88	14	185	regulation of molecular function

GOBPID stands for the Gene Ontology Biological Process identifier. P-value, odds ratio, expected and observed count are taken from the hypergeometric test for each GO term.

### Co-transcription of Genes and miRNAs

Several miRNAs are located within the coding region of a gene. These miRNAs are under the same transcriptional control as their host genes and therefore, if at all, we expect a positive regulatory effect. One does not need regression modeling to identify these miRNAs. Nevertheless, they constitute a small but good test framework for our models; we expect to find these pairs of genes and miRNAs and the resulting correlation coefficients to be positive.

Using positional information from the ensembl database (http://www.ensembl.org), we selected genes hosting one or several miRNAs and kept those pairs for which the mRNA is represented on the HGU133A microarray and the miRNA on the custom microarray used here. In unconstrained gene models in which a miRNA from the same locus as the gene served as one of the predictors, we only found positive coefficients. Likewise, in miRNA models, the coefficients of genes serving as one of the predictors for a miRNA from the same locus were always positive.

## Discussion

The NCI-60 data set is one of the most extensively studied data sets available and has emerged as a well recognized resource for cancer research and the development of computational tools. miRNA expression was first assessed by Blower [38] using custom spotted microarrays and by Gaur [39] using qPCR. Later, Liu [40], and Søkilde [41] used commercial platforms for miRNA profiling. Comparing the aforementioned platforms, Søkilde [41] found high concordance between the miRNA platforms, especially compared to a similar analysis on mRNA data of the NCI-60 data panel [42]. All analyses showed tissue specific expression of characteristic miRNAs which led to a separation of samples according to their tissue of origin in hierarchical clustering. Søkilde [41] defined tissue-specific miRNAs as the ones with significantly higher expression in the respective tissue compared to the remaining tissues. They found miR-19b tissue-specific for leukemias and colon and these two tissues of origin also show highest and second highest expression in the miRNA expression data of Blower [38], which we use.

From the perspective of target prediction, data-based regression approaches are complementary to existing sequence-based approaches. Sequence-based approaches are limited to direct interactions between miRNAs and mRNA transcripts, they might identify functionally inactive interactions, and they can not capture regulatory interplay through the co-expression of genes and miRNAs. The regression approaches on the other hand do not suffer from any of these limitations. First, it is more likely to capture functional interactions by taking expression profiles into account. Second, regression captures indirect regulation where the miRNA has an effect on an intermediate player, which then affects the mRNA levels observed. Third, regulation by mechanisms not yet described can be captured. This is advantageous from the systems biology perspective but disadvantageous from the perspective of understanding regulatory mechanisms on the level of molecular interactions. It is important to note that there is a conceptual difference between the notion of a predictor and that of a regulator. Although regression is directional in the sense that the role of predicting variables and predicted variables is different, this must not be interpreted as modeling causal interactions. Regression captures correlation of expression values, which can arise in many ways. A gene can regulate a miRNA, the miRNA can regulate the gene, or a third molecule, miRNA or gene, can regulate both of them. The picture becomes even more complicated when several molecules regulate a target in concert. While it is an advantage of the regression-based approach to be sensitive to all these forms of functional regulation, it is a disadvantage that regression can not distinguish between them. This in contrast is a strength of the sequence based target prediction approach. It builds on the molecular mechanisms of mRNA degradation at the RISC complex [43] and hence captures causal regulation mechanisms.

A general advantage of expression data-based approaches is that they are more likely to capture context specific functional interactions. miRNA mediated gene regulation can change during cell differentiation and oncogenic transformation, while sequence patterns do not. Regulation events require that a miRNA and an mRNA are expressed at the same time in the same compartment of a cell, since the miRNA must physically bind to the mRNA. This may be the case in one type of cells and not in another. Moreover all necessary proteins to form a RISC complex need to be present, which might also not be given at all times and locations. In addition, cell specific alternative splicing and polyadenylation can remove regulatory sites from the gene transcript and lead to different gene regulation, as observed in proliferating cells which have shorter 3′-UTRs with less miRNA binding sites [44]. Furthermore, examples show that miRNA-mediated repression can be altered in response to changing environmental conditions [45]. There might be even more cell specific factors influencing the interplay between miRNA and mRNA which are waiting to be discovered. In many cases, it is still unclear in which context a miRNA can functionally bind to a gene transcript. While in some contexts, all necessary factors might be present, in others, binding could be prevented because conditions are not adequate. Here lies an additional strength of the paired profiles approach. It can take cellular contexts into account by selecting gene and miRNA expression datasets from cells or tissues under different conditions. Although the NCI-60 data hold samples from many diverse cellular contexts, these subsets are too small to be analyzed separately. Nevertheless, the approach can be used once larger paired gene-miRNA data sets are available. This will allow for finding functional interactions in specific contexts.

In principle, the LARS approach shown here is very similar to the Bayesian modeling approach by [17]. These authors use gene expression profiles of mRNAs and miRNAs, plus miRNA seed sequence information in a Bayesian model. In a follow-up publication [18], they also include mRNA sequence features in the model, but this added little to the total accuracy of their predictions. Very recently, Lu et al. ([19]) proposed a LASSO regression model considering miRNA and mRNA expression profiles, miRNA binding site information, and availability of the RISC complex. They could show that the LASSO regression model is powerful in refining sequence based miRNA target predictions. However, constraining on the occurrence of a seed sequence in both approaches did not allow finding interactions based on non-canonical, not perfectly matching target site reliant, mechanisms.

In summary, we have presented a regression analysis of paired gene and miRNA profiles. We have shown that miRNA expression can predict gene expression with high accuracy and, likewise, that miRNA expression can be predicted from gene expression. Moreover, we provide lists of potential functional targets for a large set of miRNAs including both known targets with one or several miRNA binding sites, and previously unidentified targets lacking a canonical seed binding site. In almost all instances multiple miRNA predictor models yielded better predictive performances than single miRNA models. This observation suggests that miRNA mediated regulation can not be reduced to individual miRNA-mRNA interactions.

## Methods

### Data Analysis and Computational Approach

#### Data preprocessing

We used data from 59 cell lines of the NCI-60 panel for which paired mRNA and miRNA expression profiles were available. The mRNA expression raw data (Affymetrix hgu133a microarrays) was downloaded from the ArrayExpress database (http://www.ebi.ac.uk/microarray-as/ae/, accession number E-GEOD-5720) and normalized using the VSN algorithm [46]. Internal control probe sets (identifiers starting with ‘AFFX’ and probe sets mapping multiple different transcripts (ending with ‘_x_at’) were removed and remaining probe sets were mapped on to entrez identifiers. Whenever more than one probe set mapped to the same entrez identifier, the one with higher mean expression across samples was retained. This procedure yielded 12,196 probe sets targeting one entrez gene each. We then discarded probe sets that showed little variation across samples and kept those 40% of probe sets with the highest inter-quartile range (4,878 probe sets).

The miRNA data set from Blower et al. [38] was used. miRNA preprocessed expression data was also obtained from ArrayExpress, accession number E-MEXP-1029, and quantile normalized [47]. (Note: until March 2012, preprocessed data was available under the mentioned accession number. After Mar 2012, only raw data is provided. The preprocessing procedure is described in Blower et al. [38]). For the miRNA probes, a non-specific filtering step requiring at least 20 of the 59 samples to have unique measurements excluded all miRNA probes with constant expression levels across the majority of samples (443 of 627 miRNA probes remained). Whenever there was more than one probe targeting the same miRNA, the probe with the higher mean expression across samples was retained. This procedure yielded expression values for 311 different miRNAs that went into modeling. All analyzes were performed in R [48] and Bioconductor [49]. For simplicity of presentation, we describe our statistical analysis for the case of predicting gene expression from miRNA expression. Predicting miRNA expression from gene expression is done analogously.

### Least Angle Regression

We applied least angle regression [30], a computational efficient variant of linear regression with an 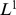-regularization term (LASSO variant, [50]). 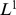-regularization does not only avoid over-fitting of high-dimensional regression models but also yields a selection of miRNA predictors, since the sum of the absolute values of the regression coefficients is constrained to be below *t*. The constraining 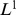-regularization can be represented by the following inequality:
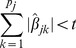
(1)


In the unconstrained models, 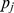 consists of all *k* miRNAs irrespective of sequence information. In the constrained models, the set of miRNAs potentially predicting a mRNA is different for each mRNA, and the set of potentially targeting miRNAs 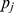, depends on mRNA *j* (response variable). Thus 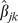 denote the fitted regression coefficients of miRNAs *k* in the set of miRNAs potentially targeting mRNA *j*, and *t* is the constraint value.

Introducing a Lagrange multiplier into linear regression, the estimate for 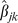 becomes:
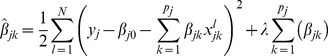
(2)


Furthermore, 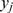 indicates the expression level of the *j*-th mRNA, 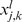 represent the expression level of the *k*-th miRNA in sample *l* potentially targeting the *j*-th mRNA.

The constraint shrinks coefficients toward zero and many of them turn out to be exactly zero, which are subsequently filtered out. The shrinking parameter 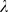 tunes the number of miRNAs remaining in the model. If it is small, many miRNAs are left. If it is large, most of them are filtered out.

For each regression model, we tuned 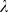 by minimizing the mean squared prediction error (MSE) in a 10-fold cross-validation. One separate model was learned for every predicted gene thus identifying a set of predictor miRNAs for it. Predictors which had entered a model are reported as candidate regulators. All predictions were merged to a predicted expression profile.

In the prevalent conception of miRNA-mediated gene regulation the miRNA is negatively regulating the expression of a gene. We reflect this in the NRM regression models by only allowing negative regression parameters except for the intercept. In view of the results of Vasudevan and coworkers [26], [27] we also ran our analysis without the parameter restriction.

Lars-LASSO regression models were implemented with the help of the R package ‘lars’ [51].

#### Integration of targeting information

In order to integrate miRNA targeting information in the regression modeling, we implemented a two step procedure. First we restricted the set of possible predictors for a specific mRNA to the miRNAs reported in a collection of miRNA target databases: TargetScan [3], version 6.1, microCosm (based on miRBase) version 5 [12], doRiNA (formerly PicTar), downloaded all miRNA targets using the web server on Mar 30, 2012 [28], and DIANA-microT, version 3.0 [29] as potential binding partners of the mRNA. All miRNAs predicted to target a specific gene by at least two of the four algorithms were taken as potential regulators, and a model was fit as discussed above. An overview over the number of interactions predicted by the four miRNA target prediciton algorithms for the genes and miRNAs of the NCI-60 dataset used in this analysis, and the overlap between the different algorithms can be found in Figure S1. For the negative constraint models, the ‘lars’ and ‘cv.lars’ functions of the ‘lars’ R package were modified analogously to the positive constraint lars described in Efron et al. [30]. In principle, when searching for the next predictor to enter the model, instead of using absolute current correlations, only negative current correlations were considered. This procedure yielded negative coefficients (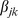) only. The code of the modified functions is provided in the supporting information section (File S7).

#### Finding interactions based on alternative regulatory mechanisms

In order to discover new regulators beyond those annotated in the miRNA target databases, we trained a second class of models on the residuals of the first. This time we used all but the miRNA probes from the first run as potential predictors. For the genes without any regulating miRNAs reported in the database, a model without targeting restrictions was fitted at this time.

#### Evaluation

Our method generates lists of candidate regulators even if the data holds no information on them. However, in this case the regression models have a very poor predictive performance. In order to evaluate predictive performance, we contrasted the observed cv-MSE with a simulated distribution of cv-MSEs that the same procedure generates if the expression profiles are not properly paired.

We did this by running our procedure on data where the samples were randomly permuted in one data set such that “paired” samples no longer stem from the same cell line. In other words, we tried to predict the expression profile of a cell line from the miRNA profile of another cell line, which can only result in random predictions. For every gene and each modeling approach, we learned 100 models with permuted pairings. For every predicted gene, we recorded the cv-MSE across the 100 permutations of each type of model and calculated an empirical p-value for the observed cv-MSE by counting the relative frequency of permutation based cv-MSEs that were smaller than the cv-MSE of the correctly paired data. Optimization of the shrinkage parameter 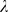 was repeated for every permutation and the optimal value could be different to the original one and varied across permutations.

We did not see major differences in p-values when comparing two sets of 100 permutations and thus limited permutations in view of the high computational cost of calculating hundreds of thousands of regression models (about 12 hours per permutation on a dual-core AMD with 2.2 GHz and 32 GiB RAM cluster node when modeling all genes).

From the distributions of p-values, False Discovery Rates (FDR) were estimated [31] and a list of top ranking models with FDR <0.01 was considered for further analysis.

### Graphical Representation of Expression Matrices

For Figures 1 (A) and 1 (B), gene expression values and gene expression values predicted with miRNA expression using the unconstrained LARS regression model were centered and scaled, and genes and samples of the gene expression data were hierarchically clustered using Manhattan distance and average linkage. The gene expression values predicted with miRNA expression (Figure 1 (B)) were plotted in the same order as the original gene expression data. The gene-wise correlation matrix of gene expression (Figure 1 (C)) was hierarchically clustered using Manhattan distance and average linkage and the correlation matrix of the gene expression values predicted with miRNA expression (Figure 1 (D)) was plotted using the same order of genes.

In Figure 3 (A), the expression values of all genes for which miR-19b-1 served as a predictor in either the direct target or the residual models were centered and scaled and then clustered with Euclidean distance and Ward linkage. The gene expression values of these genes were then predicted with either miR-19b-1 alone (Figure 3 (B)) or with all predictors included in the direct target and residual models (Figure 3 (C)).

### Over-representation of Functional Groups among Oncomir Targets

miRNA probes for the oncomirs presented in table 1 of Esquela-Kerscher [33] were selected from the NCI-60 miRNA microarray. For finding over-represented GO terms in potential oncomir targets, a conditional hypergeometric test from the Bioconductor package ‘GOstats’ [52] was applied to the targets of each individual oncomir probe. The conditional test takes into account the graph structure of Gene Ontology terms and conditions on all child nodes when testing a specific node, therefore requiring significance of the node beyond what is provided by the child nodes. P-value histograms of the conditional and the regular hypergeometric test can be found in Figure S2. Both show significance by an accumulation of low p-values, but the frequencies are lower for the conditional test because the overlap of terms has been accounted for. The ontology ‘Biological Process’ was used. GO categories with size smaller than 10 were excluded.

All 1364 genes (from 716 negative regulation and 689 residual models, minus the duplicate genes and genes without a GO term) for which we found significant prediction models served as the gene universe, conceptually representing the collection of genes from which genes could be selected to be predicted by individual miRNAs. mRNA probes had to be mapped to entrez identifiers and duplicate entrez identifiers were removed prior to the hypergeometric test.

For each oncomir probe, we considered GO terms with a p-value <0.05 as significant. The most frequently occurring GO terms over all models are summarized in Table 2. GO term enrichment analysis for the genes from negative regulation and residual models where miR-19b-1 served as a predictor was done analogously. All genes from the negative regulation and residual models served as the gene universe. Small categories (<10 genes) were not excluded.

## Supporting Information

Figure S1 **Overlap of miRNA target gene predictions.** Overlap of predicted miRNA target genes from four different prediction algorithms (microCosm/miRBase, TargetScan, DIANA/microT, PicTar/DoRiNA) Numbers show miRNA-gene pairs represented in the NCI-60 dataset used here.
(PDF)

Figure S2 **p-value histograms of GO term analysis.** p-value histograms of hypergeometric testing of oncomirs for over-representation of GO terms. Left: standard hypergeometric test, right: conditional test. Both histograms show enrichment of terms with low p-values, but the one of the conditional test has lower numbers because terms called significant because of child terms are omitted.
(PDF)

File S1 **Unconstrained gene models.** miRNA predictors and coefficients for genes with a significant unconstrained model in tabular format (csv). Coefficients were estimated from the centered and scaled miRNA expression data.
(CSV)

File S2 **Direct target gene models.** miRNA predictors and coefficients for genes with a significant DTM in tabular format (csv). Coefficients were estimated from the centered and scaled miRNA expression data.
(CSV)

File S3 **Residual gene models.** miRNA predictors and coefficients for genes with a significant residual model in tabular format (csv). Coefficients were estimated from the centered and scaled miRNA expression data.
(CSV)

File S4 **Negative regulation direct target gene models.** miRNA predictors and coefficients for genes with a significant negative regulation direct target model in tabular format (csv). Coefficients were estimated from the centered and scaled miRNA expression data.
(CSV)

File S5 **Negative regulation residual gene models.** miRNA predictors and coefficients for genes with a significant negative regulation residual model in tabular format (csv). Coefficients were estimated from the centered and scaled miRNA expression data.
(CSV)

File S6 **Unconstrained miRNA models.** Gene predictors and coefficients for miRNAs with a significant unconstrained model in tabular format (csv). Coefficients were estimated from the centered and scaled mRNA expression data.
(CSV)

File S7 **R code of all analyses.** zip archive of R scripts to compute the models and analyses as well as modified lars functions.
(ZIP)

## References

[1] Bartel DP (2009). MicroRNAs: target recognition and regulatory functions.. Cell.

[2] Bartel DP (2004). MicroRNAs: genomics, biogenesis, mechanism, and function.. Cell.

[3] Friedman RC, Farh KKH, Burge CB, Bartel DP (2009). Most mammalian mRNAs are conserved targets of microRNAs.. Genome Res.

[4] Ambros V (2004). The functions of animal microRNAs.. Nature.

[5] Pillai RS (2005). MicroRNA function: Multiple mechanisms for a tiny RNA?. RNA.

[6] Sood P, Krek A, Zavolan M, Macino G, Rajewski N (2006). Cell-type-specific signatures of microR-NAs on target mRNA expression.. PNAS.

[7] Croce CM (2009). Causes and consequences of microRNA dysregulation in cancer.. Nat Rev Genet.

[8] Brodersen P, Voinnet O (2009). Revisiting the principles of microRNA target recognition and mode of action.. Nature Rev Mol Cell Biol.

[9] Meister G, Landthaler M, Peters L, Chen PY, Urlaub H (2005). Identification of novel argonaute-associated proteins.. Curr Biol.

[10] Filipowicz W, Bhattacharyya SN, Sonenberg N (2008). Mechanisms of post-transcriptional regulation by microRNAs: are the answers in sight?. Nat Rev Genet.

[11] Betel D, Wilson M, Gabow A, Marks DS, Sander C (2008). The microRNA.org resource: targets and expression.. Nucleic Acids Res.

[12] Griffiths-Jones S, Saini HK, van Dongen S, Enright AJ (2008). miRBase: tools for microRNA genomics.. Nucleic Acids Res.

[13] Grimson A, Farh KKH, Johnston WK, Garrett-Engele P, Lim LP (2007). MicroRNA targeting specificity in mammals: determinants beyond seed pairing.. Mol Cell.

[14] Kertesz M, Iovino N, Unnerstall U, Gaul U, Segal E (2007). The role of site accessibility in mi-croRNA target recognition.. Nat Genet.

[15] Long D, Lee R, Williams P, Chan CY, Ambros V (2007). Potent effect of target structure on microRNA function.. Nat Struct Mol Biol.

[16] Elefant N, Altuvia Y, Margalit H (2011). A wide repertoire of miRNA binding sites: prediction and functional implications.. Bioinformatics.

[17] Huang JC, Morris QD, Frey BJ (2007). Bayesian inference of microRNA targets from sequence and expression data.. J Comput Biol.

[18] Huang JC, Frey BJ, Morris QD (2008). Comparing sequence and expression for predicting microRNA targets using GenMiR3.. Pacific Symposium on Biocomputing.

[19] Lu Y, Zhou Y, Qu W, Deng M, Zhang C (2011). A Lasso regression model for the construction of microRNA-target regulatory networks.. Bioinformatics.

[20] Betel D, Koppal A, Agius P, Sander C, Leslie C (2010). Comprehensive modeling of microRNA targets predicts functional non-conserved and non-canonical sites.. Genome Biol.

[21] Radfar MH, Wong W, Morris Q (2011). Computational prediction of intronic microRNA targets using host gene expression reveals novel regulatory mechanisms.. PLoS One.

[22] Ritchie W, Rajasekhar M, Flamant S, Rasko JEJ (2009). Conserved expression patterns predict microRNA targets.. PLOS Comput Biol.

[23] Stanhope SA, Sengupta S, den Boon J, Ahlquist P, Newton MA (2009). Statistical use of argonaute expression and RISC assembly in microRNA target identification.. PLoS Comput Biol.

[24] Wang YP, Li KB (2009). Correlation of expression profiles between microRNAs and mRNA targets using NCI-60 data.. BMC Genomics.

[25] John B, Sander C, Marks DS (2006). MicroRNA Protocols, Humana Press, volume 342 of Methods in Molecular Biology, chapter Prediction of Human MicroRNA Targets.. http://dx.doi.org/10.1385/1-59745-123-1:101.

[26] Vasudevan S, Tong Y, Steitz JA (2007). Switching from repression to activation: microRNAs can up-regulate translation.. Science.

[27] Vasudevan S, Tong Y, Steitz JA (2008). Cell cycle control of microRNA-mediated translation regulation.. Cell Cycle.

[28] Anders G, Mackowiak SD, Jens M, Maaskola J, Kuntzagk A (2012). doRiNA: a database of RNA interactions in post-transcriptional regulation.. Nucleic Acids Res.

[29] Maragkakis M, Reczko M, Simossis VA, Alexiou P, Papadopoulos GL (2009). DIANA-microT web server: elucidating microRNA functions through target prediction.. Nucleic Acids Res.

[30] Efron B, Hastie T, Johnstone I, Tibshirani R (2004). Least angle regression.. The Annals of Statistics.

[31] Benjamini Y, Hochberg Y (1995). Controlling the false discovery rate: a practical and powerful approach to multiple testing.. J R Statist Soc B.

[32] Slawski M, zu Castell W, Tutz G (2009). Feature selection guided by structural information. Technical report, Department of Statistics, University of Munich.. URL.

[33] Esquela-Kerscher A, Slack FJ (2006). Oncomirs - microRNAs with a role in cancer.. Nature Reviews Cancer.

[34] The Gene Ontology Consortium (2000). Gene Ontology: tool for the unification of biology.. Nature Genetics.

[35] Olive V, Bennett MJ, Walker JC, Ma C, Jiang I (2009). miR-19 is a key oncogenic component of mir-17–92.. Genes Dev.

[36] Brambillasca F, Mosna G, Colombo M, Rivolta A, Caslini C (1999). Identification of a novel molecular partner of the E2A gene in childhood leukemia.. Leukemia.

[37] Dugan KA, Wood MA, Cole MD (2002). TIP49, but not TRRAP, modulates c-Myc and E2F1 dependent apoptosis.. Oncogene.

[38] Blower PE, Verducci JS, Lin S, Zhou J, Chung JH (2007). MicroRNA expression profiles for the NCI-60 cancer cell panel.. Mol Cancer Ther.

[39] Gaur A, Jewell DA, Liang Y, Ridzon D, Moore JH (2007). Characterization of microRNA expression levels and their biological correlates in human cancer cell lines.. Cancer Res.

[40] Liu H, D’Andrade P, Fulmer-Smentek S, Lorenzi P, Kohn KW (2010). mRNA and microRNA expression profiles of the NCI-60 integrated with drug activities.. Mol Cancer Ther.

[41] Søkilde R, Kaczkowski B, Podolska A, Cirera S, Gorodkin J (2011). Global microRNA analysis of the NCI-60 cancer cell panel.. Mol Cancer Ther.

[42] Shankavaram UT, Reinhold WC, Nishizuka S, Major S, Morita D (2007). Transcript and protein expression profiles of the NCI-60 cancer cell panel: an integromic microarray study.. Mol Cancer Ther.

[43] Meister G, Tuschl T (2004). Mechanisms of gene silencing by double-stranded RNA.. Nature.

[44] Sandberg R, Neilson JR, Sarma A, Sharp PA, Burge CB (2008). Proliferating cells express mRNAs with shortened 3′ untranslated regions and fewer microRNA target sites.. Science.

[45] Bhattacharyya SN, Habermacher R, Martine U, Closs EI, FilipowiczW (2006). Relief of microRNA-mediated translational repression in human cells subjected to stress.. Cell.

[46] Huber W, von Heydebreck A, Sueltmann H, Poustka A, Vingron M (2002). Variance stabiliza-tion applied to microarray data calibration and to the quantification of differential expression.. Bioinformatics.

[47] Bolstad BM, Irizarry RA, Astrand M, Speed TP (2003). A comparison of normalization methods for high density oligonucleotide array data based on variance and bias.. Bioinformatics.

[48] R Development Core Team (2009). R: A language and environment for statistical computing.. R Foundation for Statistical Computing, Vienna, Austria.

[49] Gentleman RC, Carey VJ, Bates DM, Bolstad B, Dettling M (2004). Bioconductor: Open software development for computational biology and bioinformatics.. Genome Biology.

[50] Tibshirani R (1996). Regression shrinkage and selection via the lasso.. J Royal Statist Soc B.

[51] Hastie T, Efron B (2007). lars: Least Angle Regression, Lasso and Forward Stagewise.. http://www-stat.stanford.edu/hastie/Papers/LARS.

[52] Falcon S, Gentleman R (2007). Using GOstats to test gene lists for GO term association.. Bioinformatics.

