# Supplementary figures and images for: A Least Angle Regression Model for the Prediction of Canonical and Non-Canonical miRNA-mRNA Interactions
Source: PLoS One. 2012 Jul 17;7(7):e40634. doi: 10.1371/journal.pone.0040634 (PMC3398908; doi:10.1371/journal.pone.0040634)

# Venn Diagram

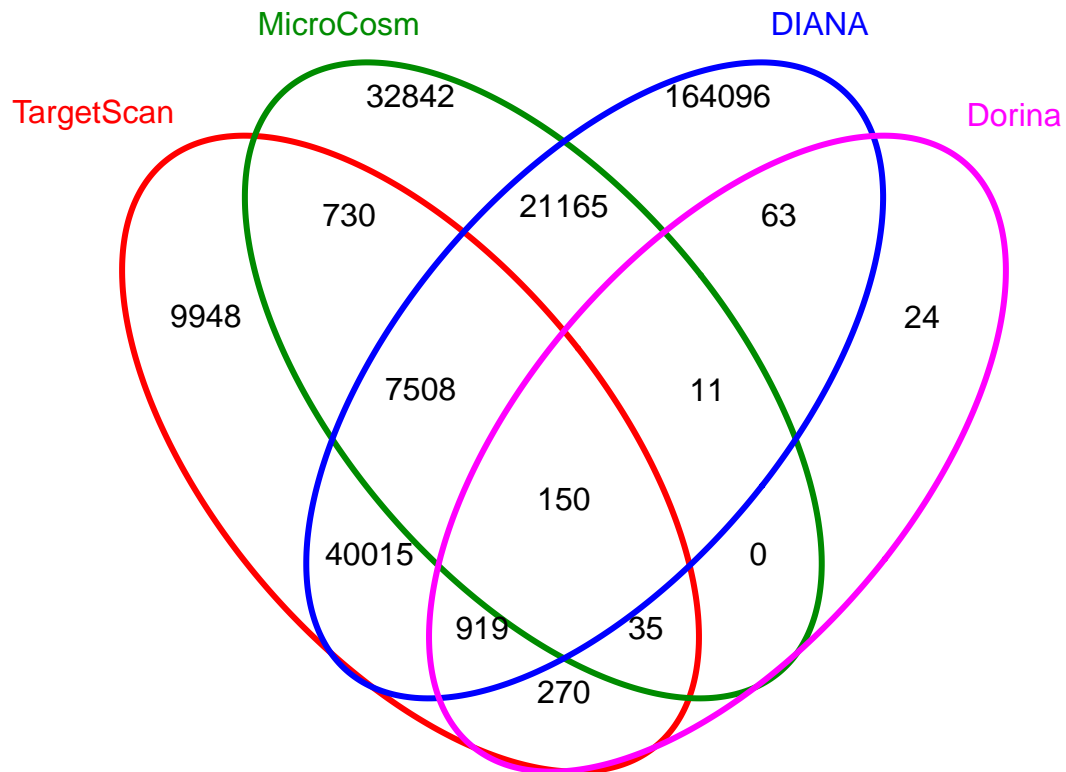

overlap of miRNA target site predictions

Supplement: Figure S1 — Overlap of miRNA target gene predictions. Overlap of predicted miRNA target genes from four different prediction algorithms (microCosm/miRBase, TargetScan, DIANA/microT, PicTar/DoRiNA) Numbers show miRNA-gene pairs represented in the NCI-60 dataset used here. (PDF) [file pone.0040634.s001.pdf]

**p-value histogram GSE**

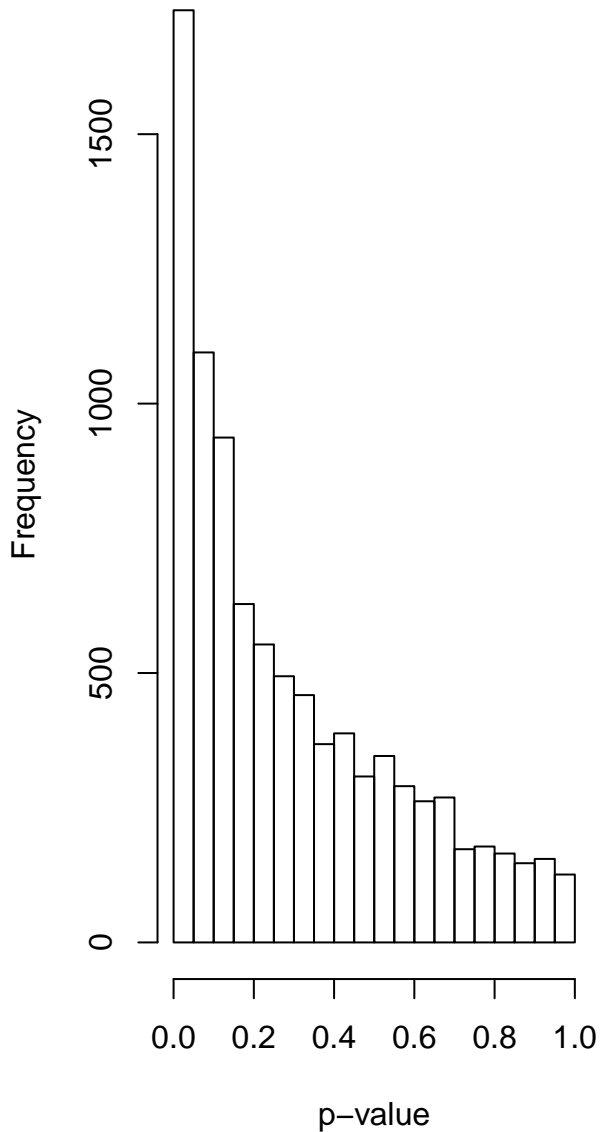

**p-value histogram GSE  
conditonal test**

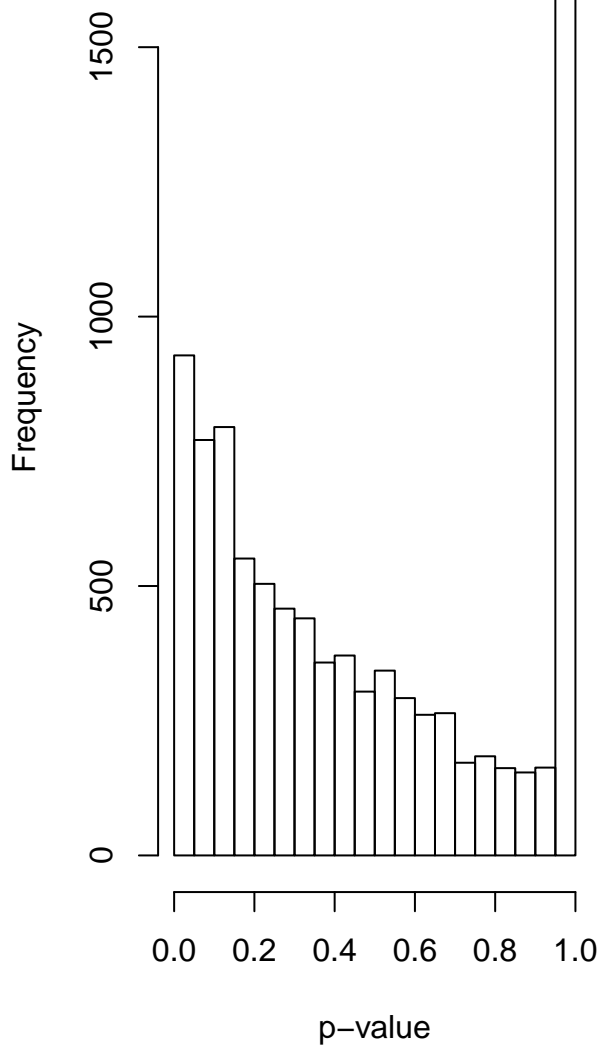

Supplement: Figure S2 — p-value histograms of GO term analysis. p-value histograms of hypergeometric testing of oncomirs for over-representation of GO terms. Left: standard hypergeometric test, right: conditional test. Both histograms show enrichment of terms with low p-values, but the one of the conditional test has lower numbers because terms called significant because of child terms are omitted. (PDF) [file pone.0040634.s002.pdf]
